# Periodontal Health, Nutrition and Anthropometry in Professional Footballers: A Preliminary Study

**DOI:** 10.3390/nu13061792

**Published:** 2021-05-25

**Authors:** João Botelho, Filipa Vicente, Laura Dias, André Júdice, Paula Pereira, Luís Proença, Vanessa Machado, Leandro Chambrone, José João Mendes

**Affiliations:** 1 Clinical Research Unit (CRU), Centro de Investigação Interdisciplinar Egas Moniz (CiiEM), Egas Moniz—Cooperativa de Ensino Superior, 2829-511 Almada, Portugal; laura.dias.faculdade@gmail.com (L.D.); ajudice@egasmoniz.edu.pt (A.J.); vmachado@egasmoniz.edu.ot (V.M.); jmendes@egasmoniz.edu.pt (J.J.M.); 2 Evidence-Based Hub, Clinical Research Unit, Centro de Investigação Interdisciplinar Egas Moniz, 2829-511 Almada, Portugal; lproenca@egasmoniz.edu.pt (L.P.); Leandro_chambrone@hotmail.com (L.C.); 3 Grupo de Estudos em Nutrição Aplicada (GENA), CiiEM, Egas Moniz—Cooperativa de Ensino Superior, 2829-511 Almada, Portugal; fvicente@egasmoniz.edu.pt (F.V.); pereira.paula1@gmail.com (P.P.); 4 Quantitative Methods for Health Research (MQIS), CiiEM, Egas Moniz—Cooperativa de Ensino Superior, 2829-511Almada, Portugal; 5 School of Dentistry, Ibirapuera University, 04661-100 São Paulo, Brazil; 6 Unit of Basic Oral Investigation (UIBO), Universidad El Bosque, 131 A-02 Bogota, Colombia

**Keywords:** periodontitis, periodontal disease, inflammation, periodontal medicine, oral health, epidemiology, sports, performance, nutrition, injury

## Abstract

Poor oral health in elite sport is a pressing issue, however little is known about the periodontal status of professional footballers. The aim of this study was to examine the prevalence of periodontitis in a group of professional footballers and its association with nutritional parameters and self-report non-traumatic injuries. Additionally, we assessed its association with anthropometric, dietary inflammatory load and self-reported muscular and/or articular injuries. Twenty-two professional footballers were evaluated at the beginning of the 2020–2021 season via full-mouth periodontal inspection, anthropometric measurements and the application of the dietary inflammatory index through a food intake measurement of 24 h dietary recall on two different days. Self-reporting non-traumatic muscular and articular injuries for the past 6 months were recorded from each athlete. Then we compared clinical measurements according to the periodontal status and we correlated age, periodontal and nutritional parameters. Overall, the prevalence of periodontitis was 40.9% and peri-implantitis was also observed. No significant differences were found regarding age or nutritional parameters according to the periodontal status. More non-traumatic muscular events in the past 6 months were found in the periodontitis group (55.6% vs. 38.4%), although the difference was non-significant. Both clinical attachment loss, periodontal pocket depth and the periodontal epithelial surface area revealed a significant moderate correlation with the percentage of fat mass, muscle mass, muscle mass index and total adipose folds. This group of professional footballers showed an alarming prevalence of periodontitis. Further studies shall examine whether periodontitis and periodontal treatment impact the performance of this group of athletes.

## 1. Introduction

Poor oral health in elite sport is a pressing issue as it negatively affects athletes in a multifactorial way [1]. Beyond the quality of life and psychosocial effects, poor oral health has been self-reported to impair athletes’ performances [2]. However, evidence on this topic is still too scarce.

In a recent cross-sectional study, caries (49.1%), erosive tooth wear (41.4%), calculus and gingival bleeding (77.0%) and periodontal pocket depth (PPD) of ≥4 mm (21.6%) were the most common conditions of elite and professional United Kingdom (UK) athletes [2]. These results have been previously reported in athletes participating in the London 2012 Olympic Games [3]. Furthermore, a representative study in professional footballers in the UK found periodontitis to be present in 5% of players, with gingivitis affecting 76.7% [4]. Nevertheless, these results were assessed using a basic periodontal examination, with the conceivable underestimation power enumerated by the authors. Thus, assessing periodontitis in a more up-to-date approach might be significant.

Moreover, nutrition embodies itself as a key element in elite sports [5]. On one hand, the athletes’ performance heavily depends on their dietary habits and behaviors. On the other hand, nutrition has an impact upon oral health, particularly dietary sugars, acidic food and drinks, and eating behaviors that increase the risk for caries and dental erosion [5]. Our group recently disclosed the potential association of dietary inflammatory load with periodontitis [6,7], and this has never been assessed in elite athletes.

With the present study, we aimed to examine the prevalence of periodontitis in a group of professional footballers. Additionally, we assessed its association with anthropometric, dietary inflammatory load and self-reported muscular and/or articular injuries.

## 2. Materials and Methods

### 2.1. Study Design and Participants

This observational study has been approved by the Egas Moniz Ethics Committee (Nº. 733) following the Declaration of Helsinki (revised in 2013) and the Strengthening the Reporting of Observational Studies in Epidemiology (STROBE) standards [8].

This study was carried out at the Sports Dentistry Department, a department from the Egas Moniz Dental Clinic (EMDC). A male football team from the Liga Portugal 2 (Portuguese second division of professional football) presented for oral evaluation for the 2020/2021, from September to October 2020. Due to confidentiality agreements the name of the club is not revealed.

To be eligible for this study, participants had to be: willing to participate having signed an informed consent; completed triage and periodontal assessment; completed nutritional assessment.

### 2.2. Variables

#### 2.2.1. Periodontal Examination

During oral assessment, the number of missing teeth, presence of caries and periodontal diagnosis were performed. Missing teeth were clinically confirmed, excluding third molars. Full-mouth periodontal examination was implemented by two calibrated examiners (J.B. and L.D) (on five non-participant patients), excluding third molars, by means of a manual periodontal probe (North Carolina, Hu-Friedy, Chicago, IL, USA).

Furthermore, dichotomous plaque index (PI), gingival recession (REC), PPD, and Bleeding on Probing (BoP) were circumferentially recorded (mesiobuccal, buccal, distobuccal, mesiolingual, lingual, and distolingual). PPD consisted on the distance from the free gingival margin to the pocket bottom. REC was the distance from the cementoenamel junction (CEJ) to free gingival margin and a negative sign was attributed if coronally gingival margin was observed. Clinical attachment loss (CAL) was computed as the algebraic sum of PPD and REC for each site. Furcation involvement (FI) was assessed using a furcation probe (N2 probe, Hu-Friedy; Chicago, Illinois, USA) in molars, and upper first premolars if applicable [9]. Tooth mobility was also assessed following [10].

The 2018 American Academy of Periodontology (AAP)/European Federation of Periodontology (EFP) periodontitis case definition was used to categorized the periodontal status [11]. That is, a periodontitis case was observed when interdental CAL in at least two non-adjacent teeth was detectable or buccal/oral CAL ≥3 mm with pocketing >3 mm was detectable [11]. Then, patients were categorized according to the staging (mild, moderate or severe/advanced) [11]. In the presence of implants, peri-implant condition was further examined classified under the 2018 AAP/EFP world workshop [12].

For each tooth, the periodontal inflamed surface area (PISA) and the periodontal epithelial surface area (PESA) was computed for each specific athlete. PISA and PESA were used as continuous variables [13,14]. These computations were performed by using Microsoft Excel, in the following steps:

- Computation of mean attachment loss (AL) and gingival recession for each tooth;
- Estimate of PESA from linear mean AL and gingival recession.
- Estimate of PISA through the multiplication of PESA by the proportion of sites around the tooth with bleeding on probing;
- Determination of the overall area, from the sum of all individual PISA and PESA scores, in mm^2^, for each participant.

Additional covariates were registered such as age and active smoking (that is, currently smoking). Self-reported medical disorders were summed among a list of possible conditions: diabetes mellitus, congestive heart failure, stroke, heart attack, asthma, coronary heart disease, angina, liver conditions, thyroid conditions, emphysema, bronchitis and cancer.

#### 2.2.2. Nutritional Examination

Height and weight were measured during clinical examination and body mass index (BMI) was computed as kg/m^2^. The athletes were weighed on a digital scale (Tanita BC-601, Amsterdam, The Netherlands) with an accuracy of 100 g and the height was measured using a millimeter-precision stadiometer (SECA, Hamburg, Germany).

Seven skinfolds were measured according to the ISAK procedure (tricipital, subscapular, bicipital, abdominal, suprailiac, crural and geminal) to estimate body density as per Whitters et al. [15] and Reilley et al. [16] protocols. Then, the Siri formula was used to compute the percentage of fat as the average of the values of the two formulas [17]. In order to estimate muscle mass, the thigh, geminal and bicipital perimeters were measured to estimate muscle mass using the Lee et al. formula [18]. Additionally, the waist–hip index was calculated as ratio of the circumference of the waist to that of the hips.

Moreover, food intake was assessed through two 24 h dietary recalls applied on two different days [19]. Next, a nutritional software (Nutrium, Braga, Portugal) assisted in the computation of energy intake, macronutrients (protein, carbohydrates and lipids), saturated, monounsaturated and polyunsaturated fatty acids and micronutrients.

These information allowed to calculate the dietary inflammatory index (DII) [20,21]. From a total list of 46 food elements, DII score was calculated using 26 food available parameters: energy (kcal), protein (gm), carbohydrate (gm), total sugar (gm), dietary fiber (gm), total fat (gm), total saturated fatty acids (gm), total monosaturated fatty acids (gm), total polyunsaturated fatty acids (gm), cholesterol (mg), vitamin E as alpha-tocopherol (mg), Eugenol, garlic, ginger, niacin, onion, saffron, saturated fat, trans fat, turmeric, green/black tea, flavan-3-ol, flavones, flavonols, flavonones, anthocyanidins, isoflavones, pepper, thyme/oregano, rosemary were not included because no information was available. For each athlete, we estimated the z-score of each food parameter, and then converted each z-score to a centered percentile. We then multiplied the centered percentile by the inflammatory standardized score [20], and all scores were summed as the DII. This score may range from strongly proinflammatory (the highest score) to strongly anti-inflammatory (the lowest score) [20].

#### 2.2.3. Muscular and Articular Injuries

The presence of muscular and/or articular injuries was self-reported by each athlete, up to 6 months prior to oral observation. Then, the stoppage time was categorized in 1–2 weeks, 3–4 weeks and more than 4 weeks.

### 2.3. Statistical Analysis

The data analysis was conducted using R. After testing for data normality and homoscedasticity, the Mann–Whitney test was used to compare continuous measures, and the chi-square test to compare categorical variables according to the periodontal status (periodontitis vs. no periodontitis). Continuous and categorical variables were represented as mean (± standard deviation (SD)) and percentage (%) and frequency/cases (n), respectively. The correlation between age, periodontal and nutritional parameters was assessed via Spearman correlation coefficient. All inferential analyses were performed for a 5% significance level.

## 3. Results

Twenty-two male footballers were included with an average of 27.7 (±5.4) years of age. The average BMI for the sample was 23.2 (±1.7) kg/m^2^ and none reported to be active smokers or to suffer from a chronic condition (Table 1). Overall, 40.9% of them had periodontitis (n = 9), the majority at stage I, but there was one case of stage II and one of stage III. Furthermore, only one athlete had one implant placed, however, this was diagnosed as a peri-implantitis case. On average, these players had 0.8 missing teeth.

The inter-examiner agreement was considered as good (0.92 and 0.87 for PPD and CAL, respectively), and the intra-examiner agreement was also considered as good (0.83 and 0.84 for PPD and CAL, respectively).

Regarding the periodontal status, we found no significant differences regarding age, nutritional parameters and most periodontal measures (Table 2). Only the percentage of sites with CAL ≥ 5 mm (*p* = 0.003), the percentage of sites with PPD ≥ 5 mm (*p* = 0.003) and BOP were significantly higher in periodontitis cases.

Next, we compared the self-reported muscle and articular injuries according to the periodontal status (Table 3). Players with periodontitis presented a higher percentage of non-traumatic muscular injuries (55.6%) than players without periodontitis (38.4%), however this difference in proportion was not found to be significant (*p* = 0.429). Similarly, players with periodontitis had a higher percentage of articular injuries (22.2%) than players without periodontitis (7.2%), albeit non-significant (*p* = 0.329).

Correlation analyses were performed between age, periodontal and nutritional parameters (Table 4). PPD and CAL showed a significant correlation with the percentage of fat mass, muscle mass, muscle mass index and total adipose folds. PESA exhibited a moderate negative significant correlation with the percentage of fat mass, muscle mass index and total adipose folds (rho = −0.590, −0.495 and −0.544, respectively) and a positive one with the muscle mass (rho = 0.537). Age was not found to be significantly correlated with any of the nutritional parameters.

## 4. Discussion

In this preliminary study of professional footballers, periodontitis was alarmingly prevalent among highly medically controlled athletes, and peri-implantitis was also a clinical finding. Furthermore, non-traumatic muscular and articular injuries were more self-reported by players with periodontitis, although non-significant. Nevertheless, we found no differences regarding anthropometric and nutritional parameters according to the periodontal status, but novel correlations have emerged particularly with PESA levels.

So far, and to the best of our knowledge, only three studies have assessed periodontal measures in professional footballers [4,22,23]. In the latter, one prospective study during three seasons in Barcelona F.C. reported high levels of gingivitis [22]. Later, in a group of UK professional footballers of the Premier league and Championship, 5% of the players had moderate-severe irreversible periodontal disease [4]. Recently, a study in young footballers, revealed an association between BOP and PPD with increased serum creatine kinase levels [23], a commonly used hallmark to purview muscle injury [24,25]. However, none of these studies have assessed players using up-to-date case definitions (EFP/AAP 2018), but rather clinical measures such as BOP or PPD, and the assessment for associations with nutritional, anthropometric items and past history of injuries are completely novel.

The existence of such a high prevalence of periodontitis is worrisome. First, this group of athletes has a thorough and continuous medical follow-up, though oral health seems somehow neglected, and this fact has been previously highlighted [22]. Second, the systemic consequences of periodontitis, for instance the leukocytosis and mild anemia [26], the increase in inflammatory mediators (CRP and cytokines) [27,28] and harmful consequences for health [29] may place these athletes at higher risk. Unfortunately, to date, the number of studies on the systemic effect of periodontitis in elite athletes is scarce and further studies are needed to clarify this topic.

Moreover, the self-report of muscular and articular injuries revealed a higher prevalence of recent injuries in the periodontitis cases. These results are interesting and may relate, for example, with the previously shown correlation of periodontal measures in markers of muscular injury [23]. Yet, the reader must bear in mind that the short sample and the lack of accurate injury measures (both muscular and articular) limit the validity of such results. Therefore, future studies are warranted to clarify if an ongoing status of periodontal inflammation and destruction is a risk factor towards muscle injuries (or vice-versa, i.e., an injury may have psychological impairment affecting motivation and self-care) and if its treatment mays mitigate such hypothetical risk.

Additionally, athletes are exposed to constant stressful conditions, which can induce parafunctions, where splints are very often used towards muscle injuries and trauma prevention during matches [30,31,32,33,34]. Most of these parafunctions may be linked to higher prevalence of periodontitis, however this shall be clarified in the future.

All in all, the main take-home message is the lack of proper periodontal care that these athletes have and the number of unknown consequences for them. Considering that prevention is key, medical staffs must advocate and support the search for oral continuous care at all times of the season.

### Strengths and Limitations

Despite the thorough clinical assessment (both for periodontal and nutritional data), and the novelty of these results, there are limitations worth discussing. The small sample included make these results as exploratory, and demand an increase in participants. This fact may be one of the reasons why several variables were non-significant despite its clear difference. Aside from that, we shall expand this research to female athletes, as periodontitis is not sex-dependent, but more frequent in men [35] as demonstrated by our group. Additionally, another shortcoming was the lack of information regarding the diagnosis of caries and its association with nutritional elements and injuries, and should be included in future research.

Additionally, the self-report of muscle and articular injuries is not sufficient enough to provide accuracy to our results, however players tend to be aware of these problems given the importance to their careers. The fact that these professional players are from a secondary division rather than a top league may be seen as a limitation, although we emphasize that these teams have less specialty diversity in their medical staff, for example due to financial restrictions, and for this reason may be at higher risk. Additionally, without having adequate oral health, most players could suddenly be picked up to a higher division and hence experience a more demanding situation, the consequences of which (whether sporting or health) are uncertain.

## 5. Conclusions

Despite the continuing medical provision that professional footballers have, this group of professional footballers showed an alarming prevalence of periodontitis. Anthropometric, dietary inflammatory load and self-reported injuries showed no association, although non-traumatic muscular injuries were more prevalent in footballers with periodontitis. Further studies will examine whether periodontitis and periodontal treatment impact the performance of this group of athletes.

## Figures and Tables

**Table 1:** Athletes’ characteristics (n = 22).

Variable	Value
Age, mean (SD)	27.7 (5.4)
Active smoking, n (%)	0 (0.0)
BMI, mean (SD)	23.2 (1.7)
Chronic condition, n (%)	0 (0.0)
DII, mean (SD)	0.20 (1.66)
Missing teeth, mean (SD)	0.8 (1.7)
Periodontal status, n (%)	
Healthy	13 (59.1)
Periodontitis	9 (40.9)
Stage I	7 (31.8)
Stage II	1 (4.5)
Stage III	1 (4.5)
Athletes with implants, n (%)	1 (4.5)
Number of implants	1
Peri-implantitis	1 (100.0)

BMI—body mass index; DII—dietary inflammatory index.

**Table 2:** Periodontal and nutritional parameters according to the periodontal status.

Variable	Periodontitis (n = 9)	No Periodontitis (n = 13)	*p*-Value
Age, mean (SD)	29.4 (5.3)	26.5 (5.3)	0.150
BMI, mean (SD) (kg/m^2^)	23.4 (1.6)	23.0 (1.7)	0.719
DII, mean (SD)	−0.4 (1.2)	0.7 (1.8)	0.327
Fat mass (%), mean (SD)	10.4 (1.6)	11.2 (2.6)	0.849
Muscle mass (kg), mean (SD)	32.8 (2.3)	34.6 (4.1)	0.944
Free fat mass (kg), mean (SD)	66.3 (4.6)	69.0 (8.0)	0.992
Muscle mass index (kg/m), mean (SD)	10.5 (0.6)	10.3 (0.6)	0.952
Adipose folds in total (mm), mean (SD)	59.0 (11.9)	64.6 (18.7)	0.865
Waist–hip index, mean (SD)	0.69 (0.04)	0.70 (0.04)	0.992
Missing teeth, mean (SD)	27.1 (1.4)	27.2 (2.0)	0.932
Mean CAL (mm), mean (SD)	2.5 (0.5)	2.2 (0.7)	0.726
Sites with CAL ≥ 5 mm (%), mean (SD)	1.8 (1.3)	0.2 (0.3)	0.004
Mean PPD (mm), mean (SD)	2.6 (0.5)	2.2 (0.7)	0.654
Sites with PPD ≥ 5 mm (%), mean (SD)	1.7 (1.3)	0.2 (0.3)	0.003
Mean BOP (%), mean (SD)	14.2 (16.6)	3.3 (3.4)	<0.001
Mean PISA (mm^2^), mean (SD)	85.6 (93.6)	23.8 (33.4)	0.401
Mean PESA (mm^2^), mean (SD)	368.0 (72.2)	336.8 (111.8)	0.904

BMI—body mass index; BOP—bleeding on probing; DII—dietary inflammatory index; CAL—clinical attachment loss; PPD—periodontal pocket depth; PISA—periodontal inflamed surface area; PESA—periodontal epithelial surface area.

**Table 3:** Muscle and articular injuries according to the periodontal status.

Variable	Periodontitis (n = 9)	No Periodontitis (n = 13)	*p*-value
Non-traumatic muscular injury, n (%)	5 (55.6)	5 (38.4)	0.429
Stoppage time, n (%)			
1–2 weeks	1 (11.1)	0 (0.0)	-
3–4 weeks	2 (22.2)	3 (23.1)	
More than 4 weeks	2 (22.2)	2 (15.4)	
Articular injury, n (%)	2 (22.2)	1 (7.2)	0.329
Stoppage time, n (%)			
1–2 weeks	1 (11.1)	0 (0.0)	-
3–4 weeks	0 (0.0)	1 (7.2)	
More than 4 weeks	1 (11.1)	0 (0.0)	

**Table 4:** Correlation between age, periodontal and nutritional parameters.

Variable	Age	CAL (mm)	PPD (mm)	BOP (%)	PISA (mm^2^)	PESA (mm^2^)
Age	-	−0.177	−0.169	−0.023	0.009	−0.302
BMI (kg/m^2^)	0.197	−0.136	−0.120	−0.345	−0.265	−0.064
DII	0.051	−0.302	−0.329	−0.485 *	−0.437	−0.041
Fat Mass (%)	0.082	−0.526 *	−0.518 *	−0.184	−0.283	−0.590 **
Muscle Mass (kg)	−0.091	0.592 *	0.591 *	−0.093	−0.061	0.537 **
Free Fat Mass (kg)	0.079	−0.085	−0.062	0.080	−0.351	0.051
Muscle Mass Index (kg/m)	−0.027	−0.490 *	−0.481 *	−0.180	−0.064	−0.495 **
Adipose Folds in Total (mm)	0.070	−0.496 *	−0.494 *	−0.159	−0.261	−0.544 **
Waist–Hip Index	−0.232	−0.064	−0.044	0.107	0.079	0.052

Spearman coefficient. *p*-value * < 0.05, ** < 0.01. BMI—body mass index; BOP—bleeding on probing; DII—dietary inflammatory index; CAL—clinical attachment loss; PPD—periodontal pocket depth; PISA—periodontal inflamed surface area; PESA—periodontal epithelial surface area.

## Data Availability

Data is accessible at the NHANES website.
